# The Association Between Sleep Disorder and Female Infertility: A Mediation Analysis of Inflammatory and Oxidative Markers

**DOI:** 10.1155/mi/4572392

**Published:** 2025-04-16

**Authors:** Qiaorui Yang, Jinfu Zhang, Zhenliang Fan

**Affiliations:** ^1^Department of Gynecology, Guanghua Hospital Affiliated to Shanghai University of Traditional Chinese Medicine, Shanghai, China; ^2^Guanghua Clinical Medical College, Shanghai University of Traditional Chinese Medicine, Shanghai, China; ^3^Department of Gynecology, Shanghai Guanghua Hospital of Integrative Medicine, Shanghai, China; ^4^Nephrology Department, The First Affiliated Hospital of Zhejiang Chinese Medical University (Zhejiang Provincial Hospital of Chinese Medicine), Zhejiang, China; ^5^Academy of Chinese Medical Science, Zhejiang Chinese Medical University, Zhejiang, China

**Keywords:** female infertility, inflammation, National Health and Nutrition Examination Survey, oxidation, sleep disorder

## Abstract

**Background:** Sleep disorder in women of reproductive age may contribute to infertility development, but there is a lack of substantial evidence linking sleep disorder to inflammation and oxidative stress, and the subsequent risk of infertility.

**Methods:** A total of 2365 women aged 18–45 years from the National Health and Nutrition Examination Survey (NHANES) were included in this analysis. Sleep disorder and infertility were assessed according to NHANES questionnaire data module. Inflammatory and oxidative biomarkers such as high-sensitivity C-reactive protein (hs-CRP), white blood cell (WBC), gamma-glutamyl transpeptidase (GGT), albumin, ferritin, and total bilirubin were derived from the laboratory data module, and systemic immune inflammation index (SII), and system inflammation response index (SIRI) were calculated based on complete blood cell counts. A sophisticated multistage sampling design and weighted multivariable adjusted regression models were employed to conduct comprehensive analysis. Mediation models were applied to explicate the mediating role of biomarkers of inflammation and oxidative stress.

**Results:** Compared to the noninfertility group, the infertile participants had a higher incidence of sleep disorder (34% vs. 25%, *p* < 0.05). In models with fully adjusted covariates, sleep disorder was positively associated with infertility risk (OR: 1.58; 95%CI: 1.01–2.50, *p* < 0.05), particularly in subgroups of individuals aged over 30 years old (OR: 1.75; 95%CI: 1.00–3.04, *p* < 0.05) or with a body mass index (BMI) ≥ 30 kg/m^2^ (OR:2.05; 95%CI: 1.00–4.22, *p* < 0.05). In terms of mechanisms, there were significant correlations between inflammatory and oxidative markers and both sleep disorder and infertility. Mediation analysis indicated that hs-CRP, SII, SIRI, GGT, and total bilirubin played a significant mediating role in the relationship between sleep disorder and infertility, accounting for 0.4822%, 6.0515%, 1.2485%, 5.1584%, and 0.4738%, respectively.

**Conclusions:** Sleep disorder is a significant risk factor for infertility, particularly in women aged >30 years or with obesity. Furthermore, the presence of inflammation and oxidative stress status in the body, which also significantly mediate the association between sleep disorder and infertility, can be swiftly and repeatedly identified through blood tests. Sleep, as a modifiable behavioral pattern, can be regarded as a new strategy to cope with infertility.

## 1. Introduction

 Infertility, broadly defined as the inability to achieve a clinical pregnancy outcome after 1 year of unprotected intercourse, is a global issue affecting approximately 8%–12% of couples of reproductive age, with a steadily increasing prevalence [1, 2]. Infertility can be caused by a variety of factors in both men and women, mainly including ovulation disorders, endometriosis, disturbed sperm function, blocked sperm transport, varicocele, and others [3]. The impact of infertility extends beyond individual couples, as it not only hampers population quality but also poses significant threats to family relationships and the whole society. Individuals facing infertility often experience a range of negative psychological and spiritual effects, including depression, anxiety, guilt, and self-blame. It is vital to comprehend the causes and consequences of infertility in order to develop effective interventions and provide support to those encountering this challenge.

Healthy sleep patterns are crucial for optimal human functioning. Increasing evidence suggests that sleep disorders, such as sleep deprivation, delayed or advanced sleep phases, inversion of sleep rhythm, and sleep disruption, are associated with impaired female reproductive function and unfavorable clinical outcomes [4–6]. Specifically, studies have found that infertile women generally report poorer sleep quality compared to their fertile counterparts, exhibiting a greater tendency for evening chronotype and higher scores on the Pittsburgh sleep quality index (PSQI) [7]. Disturbed or poor-quality sleep affects approximately one in three women struggling with infertility or undergoing adjunctive treatments for infertility [8, 9]. Furthermore, researchers have found that the sleep status prior to assisted reproductive technology (ART) treatment plays a significant role in the number and quality of oocytes retrieved, fertilization rate, and clinical pregnancy rate, ultimately impacting the ART outcome [10–12].

Sleep plays a homeostatic regulatory role in managing immune system and suppressing inflammatory signals [13]. Sleep disorder often coincides with heightened levels of inflammatory markers. For instance, evaluated levels of systemic inflammatory markers such as interleukin-6 (IL-6), C-reactive protein, and tumor necrosis factor were observed in individuals with sleep disorder [14, 15]. Moreover, sleep-deprived mice have shown rapid accumulation of proinflammatory cytokines such as IL-6 and IL-17 A, as well as chemokines like C-X-C motif chemokine ligand 1 (CXCL1) and CXCL2 in blood, ultimately leading to neutrophilia and a cytokine storm-like syndrome [16]. Melatonin, an important regulator of circadian sleep patterns also acts as an antioxidant and anti-inflammatory agent [17]. Existing evidence suggests that high levels of melatonin in follicular fluid (FF) can effectively safeguard ovarian function, maintain oocyte quality and quantity, and serve as a vital protective factor against oxidative stress and inflammatory damage in ovarian tissue [18, 19]. Similarly, infertile patients often experience overactivation of oxidative stress and inflammatory processes as well [20, 21], leading to an imbalanced FF microenvironment [22]. In addition, oxidative stress and inflammation are also implicated in infertility-related conditions such as polycystic ovary syndrome (PCOS), premature ovarian insufficiency, and endometriosis [23–25].

Building upon this knowledge, we hypothesized that oxidative stress response and inflammatory state may serve as a crucial link connecting sleep disorder and infertility. In other words, sleep disorder has the potential to disrupt the body's oxidative balance and trigger an inflammatory response, thereby promoting infertility. Therefore, the primary objective of this study is to explore the potential mediating effects and underlying biological mechanisms of the inflammatory response, measured by traditional and novel inflammatory markers such as high-sensitivity C-reactive protein (hs-CRP), white blood cell (WBC), systemic immune inflammation index (SII) and system inflammation response index (SIRI), and the oxidative stress response, represented by oxidative markers including gamma-glutamyl transpeptidase (GGT), albumin, ferritin, and total bilirubin, in the association between sleep disorder and infertility risk. Our findings aimed to contribute to the clinical management of infertility by providing valuable insights and references.

## 2. Materials and Methods

### 2.1. Study Population

Participants and relevant data were retrieved and downloaded from the National Health and Nutrition Examination Survey (NHANES, https://www.cdc.gov/nchs/nhanes/index.htm), which is a unique program of studies designed to assess and monitor the health and nutritional status of the residents of America by combining interviews and physical examinations. The survey was approved by the National Center for Health Statistics (NCHS), which is part of the Centers for Disease Control and Prevention (CDC). All the participants provided written informed consent and their privacy will be a top priority for NCHS, CDC and the survey's managing agency. The survey, which has been an ongoing project since 1999, is conducted annually on a nationally representative sample of approximately 5000 people from a random 15 counties in the United States, and the results are published biennially.

**Figure 1:**
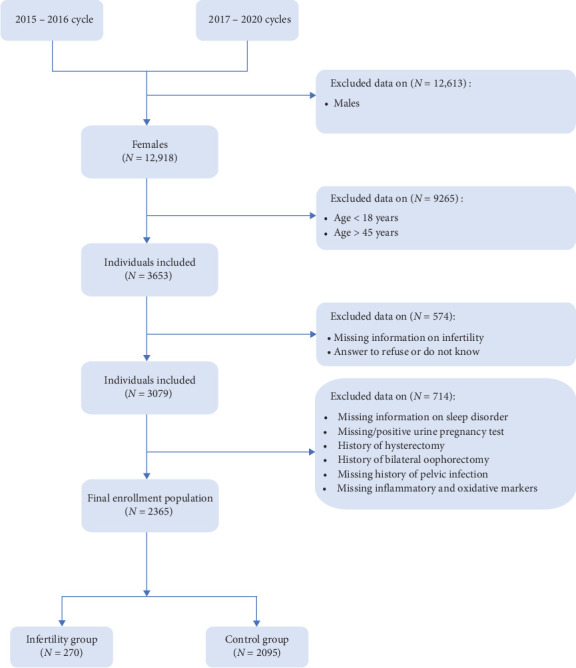
Flowchart of the study design and sample selection from the NHANES 2015 to 2020.

The subjects in present study were from 2015 to 2016 NHANES cycle and 2017–2020 NHANES cycles because it was the only period during which we had access to complete data on markers of inflammation and oxidative stress to support us in completing the analysis. A total of 12,918 women initially entered the screening process, of whom 3653 met the age requirements. Following the exclusion of individuals with incomplete infertility information, refusal to answer, or responding with “do not know,” a further 3079 women proceeded to the next stage of screening. Subsequently, patients with lacking information on sleep disorder, missing or positive urine pregnancy test results, a history of bilateral oophorectomy and/or hysterectomy, missing history of pelvic infection, or incomplete information regarding serum inflammation and oxidation markers were excluded. Ultimately, this study included 2365 patients, consisting of 270 in the infertility group and 2095 in the control group (Figure 1).

### 2.2. Outcome: Infertility

Infertility was determined based on the answers according to the “Reproductive Health” module under the “Questionnaire Data”: “RHQ076 - Have you ever been to a doctor or other medical provider because you have been unable to become pregnant?” Those who answered “Yes” were identified as infertile, while those who answered “No” were classified as part of the control group. It is important to note that participants who refused to answer or responded with “do not know” were not included the study, ensuring the accuracy and reliability of the results.

### 2.3. Exposure: Sleep Disorder

The exposure measure, sleep disorder, was assessed by the “Sleep Disorders” module under “Questionnaire Data”: “SLQ050 - Have you ever told a doctor or other health professional that you have trouble sleeping?” If the participants responded with “Yes”, they were categorized as having sleep disorder, while a response of “No” indicated normal sleeping patterns. Subjects who declined to answer or responded with “do not know” were classified as missing values.

### 2.4. Measurement of Inflammatory and Oxidative Biomarkers

There are two types of mediation variables in this study: inflammation-related markers (hs-CRP, WBC, SII, and SIRI) and oxidative stress-related markers (GGT, albumin, ferritin, and total bilirubin). The relevant laboratory test indexes were derived from modules of “Complete Blood Count with five-Part Differential—Whole Blood”, “Ferritin”, “High-Sensitivity C-Reactive Protein (hs-CRP),” and “Standard Biochemistry Profile” under “Laboratory Data”. All these tests were conducted in the NHANES mobile examination center (MEC), and serum specimens were processed, stored, and shipped to the collaborative laboratory services.

The complete blood count (CBC) was performed in duplicate on all study participants, and the Beckman Coulter® DxH 800, a quantitative and automated hematology analyzer, was applied to measure the CBC on blood specimens using venipuncture EDTA tubes and provide a distribution of all blood cells, which were obtained via the phlebotomy component. The method for measurement of ferritin was based on the cobas e601 analyzer with electrochemiluminescence immunoassay. The hs-CRP detection was performed on the Roche Cobas 6000 Chemistry Analyzer or the Beckman UniCel DxC 600/660i Analyzer. GGT, albumin, and total bilirubin were tested by Beckman Coulter UniCel DxC 800 & DxC 660i Synchron Clinical Systems or Roche Cobas 6000 Chemistry Analyzer. Internal comparative analyses conducted by NHANES staff indicated that no statistical adjustment was needed to correct for the effects of device changes. Table S1 provided a link to laboratory method files for each blood marker. All the samples were reviewed and adhered to NHANES quality assurance and quality control (QA/QC) protocols, which comply with the 1988 Clinical Laboratory Improvement Act mandates.

Based on the published literature [26, 27], SII level was calculated as platelet count *⁣*^*∗*^ neutrophil count/lymphocyte count, whereas SIRI for monocyte count *⁣*^*∗*^ neutrophil count/lymphocyte count.

### 2.5. Covariates

To further elucidate the association between sleep disorder, inflammatory and oxidative markers, and the risk of developing infertility, we included covariates that might influence infertility, including age (continuous variable), race/ethnicity (Mexican American, other Hispanic, Non-Hispanic White, Non-Hispanic Black, Non-Hispanic Asian, and other/multiracial), body mass index (BMI; underweight, normal weight, overweight, and obese), education (less than high school, high school, and more than high school), marriage (married/living with partner, widowed/divorced/separated, never married), poverty–income ratio (PIR, continuous variable), sedentary behavior (mild and severe), physical activity (light and vigorous/moderate), dietary inflammation index (DII, continuous variable), drinking status (never drinker, former drinker, and current drinker), smoking status (never smoker, former smoker, and current smoker), history of pelvic infection (yes and no), regular periods (yes and no), depression status (yes and no), cotinine (continuous variable), and calories (continuous variable) [28].

According to World Health Organization guidelines on physical activity [29, 30], vigorous/moderate activity was defined as >150 min/week of moderate-intensity or >75 min/week of vigorous-intensity physical activity, or the equivalent combination (2 min of high-intensity exercise was equivalent to 1 min of moderate-intensity aerobic activity). The sedentary behavior was categorized based on the self-reported sedentary time of the participants and defined as mild (< 360 min/day) and severe (≥ 360 min/day) [31].

Dietary inflammation index was calculated by considering 26 food parameters, and the specific calculation method was consistent with the previous published articles [32, 33]. The drinking status was determined based on the answers to questions regarding alcohol consumption in the 2015–2016 NHANES cycle, specifically, whether the individual had consumed at least 12 alcohol drinks in a year (ALQ101) or in their lifetime (ALQ110), or in the 2017–2020 NHANES cycles, whether they ever had a drink of any kind of alcohol (ALQ111) and how often they had consumed alcoholic beverages in the past 12 months (ALQ121). Smoking status was defined based on the responses to questions about smoking habits, including whether the individual had smoked at least 100 cigarettes in their lifetime (SMQ020) and whether they currently smoked cigarettes (SMQ040).

The history of pelvic infection was assessed by asking whether the individual had ever been treated for a pelvic infection or pelvic inflammatory disease (RHQ078), and regular menstrual history was determined by asking whether the individual had experienced regular periods in the past 12 months (RHQ031).

Depressive status was assessed using a nine-item depression screening instrument, where participants were asked to rate their feelings on a scale ranging from “not at all”, “several days”, “more than half the days” to “nearly every day”. The scores for each item ranged from 0 to 3. Individuals with a total score of 10 or higher were classified as having depression [34, 35].

### 2.6. Statistical Analysis

R (version 4.2.0, https://www.r-project.org/) was utilized for conducting weighted analyses in order to account for the intricate multistage sampling design employed in the NHANES. Weighted mean ± standard deviation was used to present continuous variables, and the *p* value was computed using the weighted Wilcoxon rank-sum test, which could be applied to analyze complex survey samples. Categorical variables were displayed as unweighted frequency and percentage, with the *p* value calculated using the weighted chi-squared test along with the Rao and Scott's second-order correction to address the complexities of the survey design. In addition to the comparison of baseline characteristics between the infertility group and the control group, we further performed a specific subgroup analysis considering the effect of age and BMI on infertility. Age was categorized into two subgroups, namely 18–30 years and 31–45 years. Similarly, BMI was divided into < 25 kg/m^2^, 25 to < 30 kg/m^2^, and ≥ 30 kg/m^2^ subgroups for subgroup analysis.

Subsequently, weighted multivariate logistic regression analysis was applied to elucidate the effect of sleep disorder on the risk of infertility in the overall and in subgroups by R, presented as odds ratio (OR) and 95% confidence interval (CI). Additionally, the associations between serum inflammatory (hs-CRP, WBC, SII, and SIRI) and oxidative (GGT, albumin, ferritin, and total bilirubin) markers and infertility risk were similarly analyzed using weighted logistic regression. Weighted linear regression was employed to investigate the relationships between sleep disorder and serum markers of inflammation and oxidation, with results presented as *β* value along with 95%CI. In the crude model, no adjustment for covariates was made. Model 1 was adjusted for age, BMI, race/ethnicity, education, marriage, PIR, history of pelvic infection, regular periods, depression status, calories, physical activity, and sedentary behavior. Model 2 further included the adjustments for additional covariates such as drinking status, smoking status, DII, and cotinine.

To explore the direct and indirect effects, as well as the degree of mediating effects of serum inflammatory and oxidative markers on infertility risk, mediation analysis was performed using the mediation package in R (version 4.2.0). The direct effect referred to the association between sleep disorder and the risk of infertility, while the indirect effect represented the association between sleep disorder and infertility mediated by inflammatory and oxidative markers. The mediation proportion indicated the percentage of the mediating effect over the total effect. The mediation analysis was adjusted for a comprehensive set of variables, including age, BMI, race/ethnicity, education, marriage, PIR, history of pelvic infection, regular periods, depression status, calories, physical activity, sedentary behavior, drinking status, smoking status, DII, and cotinine.

All the analyses were conducted using R (version 4.2.0), and statistical significance was considered at *p* < 0.05.

## 3. Results

### 3.1. Characteristics of the Participants

As shown in Table 1, a total of 2365 eligible participants aged 18–45 years were included in this study, comprising 270 infertile patients and 2095 noninfertile individuals, representing a weighted population of 5,106,714 and 39,435,548, respectively. The average age of the infertility group was 34 ± 6 years old, and that of the control group was 31 ± 7 years old, signifying a significant difference between the two groups (*p* < 0.001). Additionally, significant differences were observed in BMI, marriage and drinking status between the two groups (*p* < 0.05). However, there were no statistically significant differences found in race/ethnicity, education, PIR, sedentary behavior, physical activity, DII, smoking status, history of pelvic infection, history of regular periods, depression status, serum cotinine, and average daily calories intake between the infertility and control groups (*p* > 0.05). Besides, the weighted prevalence of sleep disorder in the infertility group was significantly higher than that in the noninfertility group (34% vs. 25%, *p* < 0.05).

### 3.2. Relationship Between Sleep Disorder and the Risk of Infertility

Three weighted logistic regression models with different covariates were constructed to illustrate the association between sleep disorder and the risk of infertility, as shown in Table 2. In the crude model, the risk of infertility was 1.57 times higher in people with sleep disorder compared to those with normal sleep (OR: 1.57, 95%CI: 1.04–2.39, *p* < 0.05). After adjusting for age, BMI, race/ethnicity, education, marriage, PIR, history of pelvic infection, regular periods, depression status, calories, drinking status, smoking status, physical activity, sedentary behavior, DII, and cotinine (Model 2), the trend remained stable and OR was 1.58 (95%CI: 1.01–2.50, *p* < 0.05).

### 3.3. Association of Sleep Disorder, Serum Markers, and Risk of Infertility by Age and BMI Stratification

Considering previous reports on the effects of age and weight variables on infertility [36, 37], we conducted subgroup analyses stratified by age and BMI to further explore the relationship between sleep disorder and the risk of infertility. Significant differences were observed in BMI, education level, marital status, and serum cotinine levels between the control and infertile individuals aged 18–30 years, as indicated in Table S2. However, in the age subgroup of 31–45 years, only marital status displayed a notable difference. Furthermore, among the 31–45 age subgroup, the prevalence of sleep disorder was higher in the infertile populations, accounting for 37%, which did not significantly differ in the 18–30 age subgroup. Further weighted logistic regression analysis suggested that the positive association between sleep disorder and the risk of infertility was only found in the age subgroup of 31–45 years. When adjusted for all covariates (Model 2), the OR was 1.75 and 95%CI was 1.00–3.04, with a trend *p* value of <0.05 (Table 3).

In the subgroup of individuals with a BMI <25 kg/m^2^, significant differences were observed in terms of age, marital status, DII, and regular menstrual history between the infertility participants and the controls (Table S3). However, within the 25 kg/m^2^ ≤ BMI <30 kg/m^2^ subgroup, significant differences were found in age, education level, PIR, and physical activity. Among obese individuals (BMI ≥ 30 kg/m^2^), apart from marital status and physical activity, which showed significant differences between infertility and control groups, there was a significantly higher prevalence of sleep disorder in individuals with infertility compared to the control group, at a rate of 44%. Weighted logistic regression results indicated that sleep disorder increased the risk of infertility only in the subgroup with a BMI ≥ 30 kg/m^2^ (Model 2: OR: 2.05, 95%CI: 1.00–4.22, *p* < 0.05) (Table 4).

### 3.4. Association Between Inflammatory and Oxidative Markers and Infertility

Logistic regression equations were constructed for inflammatory markers and oxidative markers to clarify their association with the risk of infertility. As demonstrated in Table 5, taking into account all relevant covariates, it was observed that for each unit increment in hs-CRP, SII, and SIRI, there was a corresponding 1.025-fold, 1.033-fold, and 1.034-fold rise in the likelihood of experiencing infertility, respectively. Furthermore, GGT was identified as a detrimental factor for infertility risk, as each unit increase in GGT level was associated with a 1.005-fold increase in the risk of infertility. On the other hand, total bilirubin seemed to act as a protective factor against infertility, showing a 0.010-fold reduction in the risk of infertility per unit increase in its level. However, despite appearing as a protective factor for infertility in partially covariate-adjusted models, ferritin's effect lost statistical significance when all covariates were included in the model.

### 3.5. Correlation of Sleep Disorder With Inflammatory and Oxidative Markers

Furthermore, we undertook an in-depth investigation into the direct associations between sleep disorder and four blood inflammatory biomarkers, as well as four oxidative biomarkers. Through the utilization of a fully adjusted regression model, our research revealed a significant positive relationship between sleep disorder and hs-CRP (*β*: 0.94, 95%CI: 0.00–1.87), SII (*β*: 54.29, 95%CI: 21.04–87.53), SIRI (*β*: 0.12, 95%CI: 0.02–0.22), and GGT (*β*: 4.95, 95%CI: 1.21–8.70) levels. Conversely, we observed a noteworthy negative correlation between sleep disorder and total bilirubin levels (*β*: −0.78, 95%CI: −1.54 to −0.01), which may be a potential protective factor (Tables 6 and 7).

### 3.6. Mediation Effect of Inflammatory and Oxidative Markers on the Association Between Sleep Disorder and Infertility

**Figure 2:**
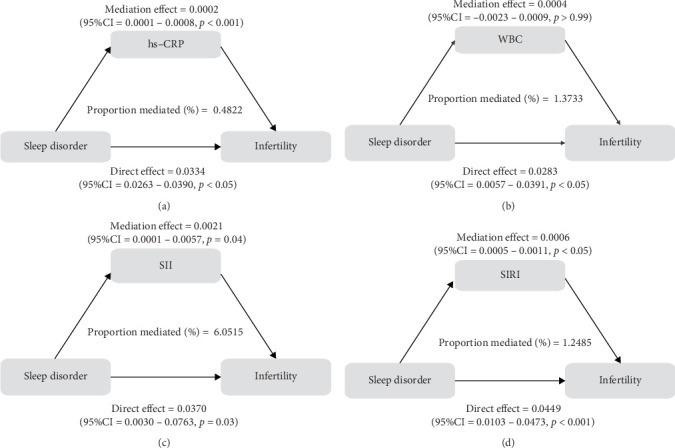
Pathway diagram of mediating analysis of inflammatory biomarkers in the relationship between sleep disorder and infertility. A–D plots represented the mediating effects of hs-CRP, WBC, SII, and SIRI in sleep disorder and infertility, respectively. CI, confidence interval; hs-CRP, high-sensitivity C-reactive protein; SII, systemic immune inflammation index; SIRI, system inflammation response index; WBC, white blood cells. (A) Mediation effect diagram for assessing the mediating effect of hs-CRP between sleep disorder and infertility; (B) Mediation effect diagram for assessing the mediating effect of WBC between sleep disorder and infertility; (C) Mediation effect diagram for assessing the mediating effect of SII between sleep disorder and infertility; (D) Mediation effect diagram for assessing the mediating effect of SIRI between sleep disorder and infertility.

**Figure 3:**
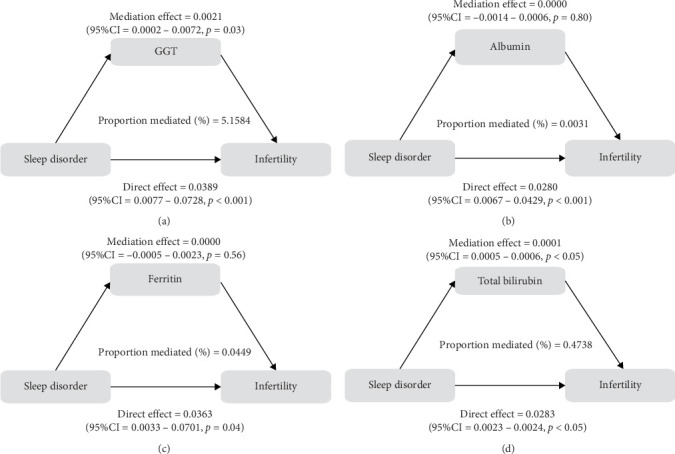
Pathway diagram of mediating analysis of oxidation biomarkers in the relationship between sleep disorder and infertility. A–D plots represented the mediating effects of GGT, albumin, ferritin, and total bilirubin in sleep disorder and infertility, respectively. CI, confidence interval; GGT, gamma-glutamyl transpeptidase. (A) Mediation effect diagram for assessing the mediating effect of GGT between sleep disorder and infertility; (B) Mediation effect diagram for assessing the mediating effect of albumin between sleep disorder and infertility; (C) Mediation effect diagram for assessing the mediating effect of ferritin between sleep disorder and infertility; (D) Mediation effect diagram for assessing the mediating effect of total bilirubin between sleep disorder and infertility.

To further validate the aforementioned hypothesis, we conducted an exploration into the potential mediation of inflammatory and oxidative markers between sleep disorder and the risk of infertility. All mediation analyses were adjusted for age, BMI, race/ethnicity, education, marriage, PIR, history of pelvic infection, regular periods, depression status, calories, physical activity, sedentary behavior, drinking status, smoking status, DII, and cotinine. We observed statistically significant mediating effects when hs-CRP, SII, SIRI, GGT, and total bilirubin were included in the mediation analysis of the association between sleep disorder and infertility, which explained 0.4822%, 6.0515%, 1.2485%, 5.1584%, and 0.4738% of the association, respectively (Figures 2 and 3). Moreover, WBC, albumin, and ferritin explained 1.3733%, 0.0031%, and 0.0449% of this association, respectively. Despite the direct impact being significant in all cases, their mediating effects were not statistically significant (Figures 2 and 3).

## 4. Discussion

This study, based on the NHANES database, is the first to delve into the mediating roles of inflammatory response (hs-CRP, WBC, SII, and SIRI) and oxidative stress (GGT, albumin, ferritin, and total bilirubin) in the association between sleep disorder and infertility risk. The findings of our study indicated that infertile women of childbearing age (18–45 years old) were more likely to have sleep disorder, which was positively correlated with the risk of infertility. Specifically, further stratified by age and BMI, sleep disorder emerged as an independent risk factor for infertility in the subgroup of women aged 31–45 years or with a BMI of ≥ 30 kg/m^2^. A similar observation was made by Yao et al., [38] who noted a reduced number of MII oocytes, two pronuclei and good-quality embryos in women with subjective sleep disorder. In particular, sleep duration exhibited a positive correlation with fertilization rate, embryo quality, implantation rate, and clinical pregnancy rate in women over 30 years old but not in those under 30 years old [38]. Sleep characteristics were also found to be predictive of ART outcomes in populations with infertility [10]. Additionally, there is more clinical evidence supporting the connection between sleep disorder and disturbances in reproductive hormones such as luteinizing hormone (LH), estradiol, and testosterone [39], as well as irregular menstrual cycle, heavy menstrual bleeding, prolonged menstrual flow, period pain, and premenstrual syndrome [40, 41]. Reduced fecundability ratios have also been observed, further highlighting the link between sleep disorder and reproductive system diseases [42]. Animal models have further directly demonstrated that sleep disorder patterns, like shift work, can impair the occurrence of pre-ovulation LH surge, leading to reduced fertility outcomes [43].

Obstructive sleep apnea (OSA), a common pattern of sleep disorder, often coexists with obesity. Studies have revealed that sleep disorder, such as insufficient sleep and circadian rhythm disruptions, can contribute to weight gain [44], while abdominal fat accumulation and a large neck circumference further become risk factors for OSA [45], which in turn induce sleep disorder. And being overweight can directly lead to poor sleep [46]. In addition to the aforementioned effects, obesity also has direct detrimental effects on the reproductive system, including precocious puberty, ovarian dysfunction, and even infertility [47, 48]. Mokhlesi et al. observed a higher prevalence of obesity and OSA in individuals with PCOS, and the risk of developing OSA was found to be associated with severe obesity [49], supporting previous findings that higher BMI is linked to the occurrence of OSA in the PCOS population [50, 51]. To summarize, obesity can either trigger or worsen sleep disorder, and vice versa. Both factors, either individually or in combination, can eventually significantly impact reproductive function.

This study also revealed that individuals with elevated levels of hs-CRP, SII, SIRI, and GGT were at an increased risk of experiencing infertility. Conversely, total bilirubin was found to be a protective factor against infertility. SII and SIRI, which are novel assessment parameters for measuring systemic inflammation levels, provide a comprehensive evaluation of the immune and inflammatory status of the body. However, there is currently a lack of research investigating the correlation between SII and SIRI and infertility. This cross-sectional study, conducted on a representative population, presents evidence suggesting that high SII and SIRI levels may serve as candidate indicators reflecting elevated inflammation that may induce infertility in the state of sleep disorders.

As a nonspecific marker of systemic inflammatory response, hs-CRP is synthesized by the liver. Studies have found an elevation in the levels of hs-CRP among PCOS patients, particularly those with OSA [50, 52], which indicated that OSA might exacerbate the inflammatory response within the body. Bilirubin, an essential regulator of various biological functions, has been observed to undergo changes in the FF of infertile women. This alteration was associated with oxidative/nitrosative stress changes, activation of defense mechanisms and an increase in bilirubin levels [22], and the total bilirubin levels demonstrated a negative correlation with the number of fertilized eggs, blastocysts, high-quality blastocysts, as well as clinical pregnancy and birth rates [22]. These findings suggest that oxidative stress in the microenvironment of follicle growth and development plays a role in adverse reproductive outcomes. Additionally, infertile women exhibited lower levels of ferritin [53], with <30 *µ*g/L being considering to have clinical predictive value in unexplained infertility [54]. Albumin, known for its osmoregulatory and antioxidant properties, has been found to be reduced in FF among infertile individuals, including those with reduced ovarian reserve, endometriosis, and idiopathic infertility, and has a positive impact on reproduction, suggesting that albumin in FF serves as a crucial buffer against oxidative conditions [55]. However, in this study, we were unable to establish a connection between albumin, ferritin, sleep disorder and infertility. This may be attributed to the low specificity and sensitivity of ferritin and albumin when measured in peripheral blood.

Similar to our findings, several studies have reported associations between sleep disorder and biomarkers of inflammation and oxidative stress. Among OSA patients, albumin, neutrophil count, monocyte count, and SIRI were observed to be independently associated with lower oxygen saturation [56], hinting that the oxidative stress response under hypoxia may be involved. Similarly, in a study conducted by Güneş et al., it was observed that OSA patients had higher SII, which could be a rapid and convenient biomarker for OSA prediction with a sensitivity of 84.7% and a specificity of 29.5% when a cutoff value of 290 was used [57]. Kim et al. [58] found that PSQI was independently associated with SII in severe OSA patients. Additionally, high levels of GGT and ferritin have been linked to the severity of OSA [59, 60], implying the attenuation of antioxidant defenses [61]. These findings highlight the role of oxidative stress and inflammation in sleep disorder, containing OSA. Furthermore, through mediation analysis, we discovered significant regulatory roles of hs-CRP, SII, SIRI, GGT, and total bilirubin in sleep disorder and infertility, which strongly supports the hypothesis that sleep disorder may increase the risk of infertility by inducing oxidative stress and inflammation, providing important clues and evidence for understanding the potential mechanisms underlying the relationship between sleep disorder and infertility.

On the other hand, exogenous supplementation of sleep-related hormones such as melatonin can effectively address the oxidative imbalance in the follicle, increase the number of oocytes, mature oocytes, and high-quality embryos during ART, and finally improve the clinical pregnancy rate [62, 63]. The above evidence highlights from another perspective that the involvement of sleep disorder in the pathological process of infertility is closely linked to oxidative stress and inflammatory response.

This study has certain limitations. First of all, due to the design characteristics of NHANES, the assessment methods of sleep disorder and infertility were based on self-reports of patients, which introduces a certain degree of subjective judgment compared with objective examination methods. Second, limited by the data collected in the NHANES database, our study did not take into account the effect of complementary treatments such as melatonin on infertility. Third, while the mediating effects of certain markers of inflammation and oxidative stress were found to be statistically significant, it is important to recognize that the magnitude of these effects were relatively small. This may be attributed to the limited sample size, which could have affected the statistical power. Therefore, it is essential to interpret the results with caution.

## 5. Conclusion

In conclusion, first of all, this study found that the increased risk of infertility was associated with sleep disorder, especially in women aged over 30 years or those who are obese. Secondly, inflammation (hs-CRP, SII, and SIRI) and oxidative stress (GGT and total bilirubin) mediated the pathological process between sleep disorder and infertility, and blood test, as a simple and efficient detection method, can effectively provide accurate and reproducible information about the body's inflammation and oxidative stress status. Sleep behavior, as a modifiable behavioral factor, provides a new strategy for coping with female infertility.

## Figures and Tables

**Table 1:** Survey-weighted characteristics of the participants by self-reported infertility.

Variables	Total (*N* = 44,542,262)	Control (*N* = 39,435,548)	Infertility (*N* = 5,106,714)	*p* Value
Age (years)	31 ± 7	31 ± 7	34 ± 6	**<0.001**
Race/ethnicity	—	—	—	0.87
Mexican American	396 (12%)	347 (12%)	49 (14%)	—
Other Hispanic	257 (8%)	225 (8%)	32 (8%)	—
Non-Hispanic White	695 (55%)	611 (55%)	84 (54%)	—
Non-Hispanic Black	600 (14%)	537 (14%)	63 (14%)	—
Non-Hispanic Asian	295 (7%)	265 (7%)	30 (5%)	—
Other/multiracial	122 (4%)	110 (4%)	12 (5%)	—
BMI (kg/m^2^)	—	—	—	**0.02**
Underweight (<18.5)	66 (2%)	61 (3%)	5 (1%)	—
Normal weight (18.5 to <25)	718 (34%)	646 (35%)	72 (26%)	—
Overweight (25 to <30)	562 (24%)	515 (24%)	47 (19%)	—
Obese (30 or greater)	1005 (40%)	859 (38%)	146 (54%)	—
Education	—	—	—	0.33
Less than high school	331 (9%)	290 (8%)	41 (12%)	—
High school	471 (21%)	414 (21%)	57 (21%)	—
More than high school	1563 (70%)	1391 (71%)	172 (66%)	—
Marriage	—	—	—	**<0.001**
Married/living with partner	1310 (57%)	1114 (55%)	196 (75%)	—
Widowed/divorced/separated	214 (9%)	184 (8%)	30 (11%)	—
Never married	841 (34%)	797 (37%)	44 (14%)	—
PIR	2.72 ± 1.67	2.70 ± 1.68	2.82 ± 1.62	0.30
Sedentary behavior	—	—	—	0.78
Mild	1612 (68%)	1439 (68%)	173 (67%)	—
Severe	753 (32%)	656 (32%)	97 (33%)	—
Physical activity	—	—	—	0.78
Light	1612 (68%)	1439 (68%)	173 (67%)	—
Vigorous/moderate	753 (32%)	656 (32%)	97 (33%)	—
DII	0.58 ± 2.00	0.54 ± 2.01	0.89 ± 1.92	0.06
Drinking status	—	—	—	**0.04**
Never drinker	938 (37%)	835 (37%)	103 (37%)	—
Former drinker	732 (28%)	629 (26%)	103 (36%)	—
Current drinker	695 (36%)	631 (37%)	64 (27%)	—
Smoking status	—	—	—	0.49
Never smoker	1692 (69%)	1517 (69%)	175 (64%)	—
Former smoker	273 (14%)	240 (13%)	33 (15%)	—
Current smoker	400 (18%)	338 (17%)	62 (21%)	—
History of pelvic infection	—	—	—	0.11
Yes	116 (4%)	93 (4%)	23 (7%)	—
No	2236 (96%)	1990 (96%)	246 (93%)	—
Regular periods	—	—	—	0.10
Yes	2222 (94%)	1970 (95%)	252 (91%)	—
No	143 (6%)	125 (5%)	18 (9%)	—
Depression status	—	—	—	0.20
Yes	242 (11%)	203 (10%)	39 (14%)	—
No	2120 (89%)	1889 (90%)	231 (86%)	—
Cotinine (ng/mL)	39 ± 98	38 ± 96	47 ± 107	0.69
Calories (kcal/d)	1844 ± 641	1847 ± 637	1820 ± 669	0.68
Sleeplessness	—	—	—	**0.03**
Yes	548 (26%)	462 (25%)	86 (34%)	—
No	1817 (74%)	1633 (75%)	184 (66%)	—

*Note:* Continuous variables were presented as weighted mean ± standard deviation and categorical variables were presented as unweighted frequency and percentage. *p* Value in bold indicated statistical significance.

Abbreviations: BMI, body mass index; DII, dietary inflammation index; PIR, poverty–income ratio.

**Table 2:** Relationship between sleep disorder and infertility.

	Crude model		Model 1		Model 2	
	OR (95% CI)	*p* Value	OR (95% CI)	*p* Value	OR (95% CI)	*p* Value
Normal	Reference		Reference		Reference	
Sleep disorder	1.57 (1.04, 2.39)	0.03	1.33 (0.76, 2.35)	0.30	1.58 (1.01, 2.50)	<0.05

*Note:* Crude model was not adjusted for any covariates. Model 1 was adjusted for age, BMI, race/ethnicity, education, marriage, PIR, history of pelvic infection, regular periods, depression status, calories, physical activity, and sedentary behavior. Model 2 was adjusted for age, BMI, race/ethnicity, education, marriage, PIR, history of pelvic infection, regular periods, depression status, calories, physical activity, sedentary behavior, drinking status, smoking status, DII, and cotinine.

Abbreviations: CI, confidence interval; OR, odds ratio.

**Table 3:** Relationship between sleep disorder and the risk of infertility based on age subgroup analysis.

	18–30 years		31–45 years	
	OR (95%CI)	*p* Value	OR (95%CI)	*p* Value
Crude model	1.22 (0.43, 3.51)	0.70	1.67 (1.01, 2.76)	<0.05
Model 1	1.11 (0.41, 2.99)	0.83	1.53 (0.82, 2.86)	0.17
Model 2	1.04 (0.42, 2.53)	0.93	1.75 (1.00, 3.04)	<0.05

*Note:* Crude model was not adjusted for any covariates. Model 1 was adjusted for BMI, race/ethnicity, education, marriage, PIR, history of pelvic infection, regular periods, depression status, calories, physical activity, and sedentary behavior. Model 2 was adjusted for BMI, race/ethnicity, education, marriage, PIR, history of pelvic infection, regular periods, depression status, calories, physical activity, sedentary behavior, drinking status, smoking status, DII, and cotinine.

Abbreviations: CI, confidence interval; OR, odds ratio.

**Table 4:** Relationship between sleep disorder and the risk of infertility based on BMI subgroup analysis.

	<25 kg/m^2^		25 to <30 kg/m^2^		≥ 30 kg/m^2^	
	OR (95%CI)	*p* Value	OR (95%CI)	*p* Value	OR (95%CI)	*p* Value
Crude model	1.20 (0.43, 3.39)	0.72	0.93 (0.32, 2.76)	0.90	1.83 (1.03, 3.23)	0.04
Model 1	1.18 (0.31, 4.47)	0.80	0.36 (0.13, 1.00)	<0.05	1.93 (1.01, 3.71)	0.04
Model 2	1.26 (0.39, 4.03)	0.68	0.50 (0.19, 1.30)	0.14	2.05 (1.00, 4.22)	0.04

*Note:* Crude model was not adjusted for any covariates. Model 1 was adjusted for age, race/ethnicity, education, marriage, PIR, history of pelvic infection, regular periods, depression status, calories, physical activity, and sedentary behavior. Model 2 was adjusted for age, race/ethnicity, education, marriage, PIR, history of pelvic infection, regular periods, depression status, calories, physical activity, sedentary behavior, drinking status, smoking status, DII, and cotinine.

Abbreviations: CI, confidence interval; OR, odds ratio.

**Table 5:** The associations between inflammatory and oxidative markers and infertility.

	Crude model	Model 1	Model 2
Inflammatory markers			
hs-CRP (mg/L)	1.018 (0.997, 1.039)	1.005 (0.979, 1.033)	**1.025 (1.000, 1.050)**
WBC (1000 cells/uL)	1.057 (0.978, 1.141)	1.000 (0.933, 1.134)	1.025 (0.929, 1.132)
SII	1.000 (1.000, 1.001)	1.030 (0.996, 1.000)	**1.033 (1.005, 1.061)**
SIRI	**1.060 (1.033, 1.088)**	**1.032 (1.006, 1.060)**	**1.034 (1.007, 1.062)**
Oxidative markers			
GGT (IU/L)	**1.007 (1.003, 1.011)**	**1.005 (1.000, 1.009)**	**1.005 (1.000, 1.010)**
Albumin (g/L)	0.951 (0.898, 1.007)	0.991 (0.920, 1.068)	0.996 (0.921, 1.076)
Ferritin (ug/L)	1.000 (0.997, 1.003)	**0.999 (1.006, 1.059)**	0.999 (0.995, 1.003)
Total bilirubin (umol/L)	0.972 (0.933, 1.012)	0.986 (0.944, 1.030)	**0.990 (1.006, 1.059)**

*Note:* The models were expressed as OR (95%CI) with bold indicating statistical significance. Crude model was not adjusted for any covariates. Model 1 was adjusted for age, BMI, race/ethnicity, education, marriage, PIR, history of pelvic infection, regular periods, depression status, calories, physical activity, and sedentary behavior. Model 2 was adjusted for age, BMI, race/ethnicity, education, marriage, PIR, history of pelvic infection, regular periods, depression status, calories, physical activity, sedentary behavior, drinking status, smoking status, DII, and cotinine.

Abbreviations: GGT, *γ*-glutamyl transferase; hs-CRP, hypersensitive C-reactive protein; SII, systemic immune inflammation index; SIRI, systemic inflammatory response index; WBC, white blood cell.

**Table 6:** The associations between sleep disorder and inflammatory markers.

	hs-CRP (mg/L)	WBC (1000 cells/uL)	SII	SIRI
Crude model	**0.93 (0.05, 1.80)**	**0.38 (0.03, 0.72)**	**51.70 (17.62, 85.78)**	**0.11 (0.01, 0.21)**
Model 1	0.41 (−0.59, 1.41)	0.22 (−0.16, 0.60)	**52.39 (18.79, 85.98)**	**0.12 (0.01, 0.22)**
Model 2	**0.94 (0.00, 1.87)**	0.25 (−0.12, 0.62)	**54.29 (21.04, 87.53)**	**0.12 (0.02, 0.22)**

*Note:* The models were expressed as *β* (95%CI) with bold indicating statistical significance. Crude model was not adjusted for any covariates. Model 1 was adjusted for age, BMI, race/ethnicity, education, marriage, PIR, history of pelvic infection, regular periods, depression status, calories, physical activity, and sedentary behavior. Model 2 was adjusted for age, BMI, race/ethnicity, education, marriage, PIR, history of pelvic infection, regular periods, depression status, calories, physical activity, sedentary behavior, drinking status, smoking status, DII, and cotinine.

Abbreviations: hs-CRP, hypersensitive C-reactive protein; SII, systemic immune inflammation index; SIRI, systemic inflammatory response index; WBC, white blood cell.

**Table 7:** The associations between sleep disorder and oxidative markers.

	GGT (IU/L)	Albumin (g/L)	Ferritin (ug/L)	Total bilirubin (umol/L)
Crude model	**6.30 (3.04, 9.55)**	−0.45 (−1.02, 0.12)	5.28 (−1.18, 11.75)	**−0.92 (−1.58, −0.25)**
Model 1	**4.50 (0.92, 8.08)**	−0.17 (−0.74, 0.39)	4.38 (−1.82, 10.58)	**−0.78 (−1.55, −0.02)**
Model 2	**4.95 (1.21, 8.70)**	−0.10 (−0.67, 0.47)	4.16 (−1.93, 10.26)	**−0.78 (−1.54, −0.01)**

*Note:* The models were expressed as *β* (95%CI) with bold indicating statistical significance. Crude model was not adjusted for any covariates. Model 1 was adjusted for age, BMI, race/ethnicity, education, marriage, PIR, history of pelvic infection, regular periods, depression status, calories, physical activity, and sedentary behavior. Model 2 was adjusted for age, BMI, race/ethnicity, education, marriage, PIR, history of pelvic infection, regular periods, depression status, calories, physical activity, sedentary behavior, drinking status, smoking status, DII, and cotinine.

Abbreviation: GGT, gamma-glutamyl transpeptidase.

## Data Availability

The datasets used and/or analyzed during the current study are available in the NHANES repository, https://www.cdc.gov/nchs/nhanes/.

## Supporting Information

Supporting Information Table S1: URLs of detailed laboratory measurement of inflammatory and oxidative markers from the NHANES survey in present study. Table S2: Characteristics of the participants based on age subgroup analysis. Table S3: Characteristics of the participants based on BMI subgroup analysis.
