# Supplementary material for: The Association Between Sleep Disorder and Female Infertility: A Mediation Analysis of Inflammatory and Oxidative Markers
Source: Mediators Inflamm. 2025 Apr 16;2025:4572392. doi: 10.1155/mi/4572392 (PMC12017959; doi:10.1155/mi/4572392)
Supplement: Supporting Information — Table S1: URLs of detailed laboratory measurement of inflammatory and oxidative markers from the NHANES survey in present study. Table S2: Characteristics of the participants based on age subgroup analysis. Table S3: Characteristics of the participants based on BMI subgroup analysis. [file 4572392.f1.docx]

Table S1. URLs of detailed laboratory measurement of inflammatory and oxidative markers from the NHANES survey in present study.

| Cycles | Laboratory measurement | URLs |
| --- | --- | --- |
| 2015-2016 | hs-CRP | https://wwwn.cdc.gov/nchs/data/nhanes/2015-2016/labmethods/HSCRP_I_MET.pdf |
|  | CBC | https://wwwn.cdc.gov/nchs/data/nhanes/2015-2016/labmethods/CBC_I_MET_Complete_Blood_Count.pdf |
|  | GGT | https://wwwn.cdc.gov/nchs/data/nhanes/2015-2016/labmethods/BIOPRO_I_MET_GGT_DXC800and660i.pdf |
|  | Albumin | https://wwwn.cdc.gov/nchs/data/nhanes/2015-2016/labmethods/BIOPRO_I_MET_ALBUMIN_DXC660i.pdf |
|  | Ferritin | https://wwwn.cdc.gov/nchs/data/nhanes/2015-2016/labmethods/FERTIN_I_MET.pdf |
|  | Total bilirubin | https://wwwn.cdc.gov/nchs/data/nhanes/2015-2016/labmethods/BIOPRO_I_MET_BILIRUBIN_DXC800and660i.pdf |
| 2017-2020 | hs-CRP | https://wwwn.cdc.gov/nchs/data/nhanes/2017-2018/labmethods/HSCRP-J-MET-508.pdf  https://wwwn.cdc.gov/nchs/data/nhanes/2019-2020/labmethods/HSCRP-K-MET-508.pdf |
|  | CBC | https://wwwn.cdc.gov/nchs/data/nhanes/2017-2018/labmethods/CBC-J-MET-508.pdf  https://wwwn.cdc.gov/nchs/data/nhanes/2019-2020/labmethods/CBC-K-MET-508.pdf |
|  | GGT | https://wwwn.cdc.gov/nchs/data/nhanes/2017-2018/labmethods/BIOPRO-J-MET-GGT-508.pdf |
|  | Albumin | https://wwwn.cdc.gov/nchs/data/nhanes/2017-2018/labmethods/BIOPRO-J-MET-Albumin-508.pdf |
|  | Ferritin | https://wwwn.cdc.gov/nchs/data/nhanes/2017-2018/labmethods/FERTIN-J-MET-508.pdf  https://wwwn.cdc.gov/nchs/data/nhanes/2019-2020/labmethods/FERTIN-K-MET-508.pdf |
|  | Total bilirubin | https://wwwn.cdc.gov/nchs/data/nhanes/2017-2018/labmethods/BIOPRO-J-MET-Total-Bilirubin-508.pdf |

Abbreviations: hs-CRP: high sensitivity C-reactive protein; CBC: complete blood count; GGT: gamma-Glutamyl transferase;

Table S2. Characteristics of the participants based on age subgroup analysis.

| **Variables** | **18-30 years** | | | **31-45 years** | | |
| --- | --- | --- | --- | --- | --- | --- |
|  | **Control**  **(N = 19,717,268)** | **Infertility**  **(N = 1,568,186)** | ***P* value** | **Control**  **(N = 19,718,280)** | **Infertility**  **(N = 3,538,528)** | ***P* value** |
| **Race/Ethnicity** |  |  | 0.63 |  |  | 0.95 |
| Mexican American | 138 (12%) | 19 (18%) |  | 209 (12%) | 30 (12%) |  |
| Other Hispanic | 97 (8%) | 9 (11%) |  | 128 (8%) | 23 (7%) |  |
| Non-Hispanic White | 308 (55%) | 24 (49%) |  | 303 (54%) | 60 (57%) |  |
| Non-Hispanic Black | 239 (14%) | 20 (12%) |  | 298 (14%) | 43 (15%) |  |
| Non-Hispanic Asian | 121 (7%) | 4 (4%) |  | 144 (7%) | 26 (5%) |  |
| Other/multiracial | 61 (4%) | 2 (6%) |  | 49 (4%) | 10 (4%) |  |
| **BMI (kg/m^2^)** |  |  | **0.01** |  |  | 0.37 |
| Underweight (<18.5) | 42 (4%) | 2 (1%) |  | 19 (2%) | 3 (2%) |  |
| Normal weight (18.5 to <25) | 359 (41%) | 19 (20%) |  | 287 (30%) | 53 (28%) |  |
| Overweight (25 to <30) | 220 (22%) | 13 (20%) |  | 295 (26%) | 34 (19%) |  |
| Obese (30 or greater) | 340 (33%) | 44 (59%) |  | 519 (42%) | 102 (51%) |  |
| **Education** |  |  | **0.02** |  |  | 0.41 |
| Less than high school | 87 (6%) | 11 (13%) |  | 203 (11%) | 30 (12%) |  |
| High school | 208 (23%) | 28 (40%) |  | 206 (18%) | 29 (13%) |  |
| More than high school | 669 (71%) | 39 (47%) |  | 722 (70%) | 133 (75%) |  |
| **Marriage** |  |  | **<0.05** |  |  | **0.02** |
| Married/Living with partner | 403 (43%) | 54 (71%) |  | 711 (67%) | 142 (77%) |  |
| Widowed/Divorced/Separated | 31 (3%) | 3 (2%) |  | 153 (13%) | 27 (15%) |  |
| Never married | 530 (54%) | 21 (27%) |  | 267 (20%) | 23 (8%) |  |
| **PIR** | 2.59 ± 1.63 | 2.13 ± 1.34 | 0.11 | 2.81 ± 1.71 | 3.10 ± 1.64 | 0.15 |
| **Sedentary behavior** |  |  | 0.19 |  |  | 0.20 |
| Mild | 642 (66%) | 56 (76%) |  | 797 (70%) | 117 (62%) |  |
| Severe | 322 (34%) | 22 (24%) |  | 334 (30%) | 75 (38%) |  |
| **Physical activity** |  |  | 0.27 |  |  | 0.41 |
| Light | 522 (49%) | 56 (61%) |  | 738 (56%) | 122 (53%) |  |
| Vigorous/Moderate | 442 (51%) | 22 (39%) |  | 393(44%) | 70(47%) |  |
| **DII** | 0.71 ± 1.96 | 1.36 ± 1.68 | 0.09 | 0.37 ± 2.04 | 0.68 ± 1.98 | 0.13 |
| **Drinking status** |  |  | 0.16 |  |  | 0.08 |
| Never drinker | 411 (38%) | 26 (27%) |  | 424 (36%) | 77 (41%) |  |
| Former drinker | 246 (23%) | 26 (36%) |  | 383 (30%) | 77 (36%) |  |
| Current drinker | 307 (39%) | 26 (37%) |  | 324 (34%) | 38 (23%) |  |
| **Smoking status** |  |  | 0.21 |  |  | 0.98 |
| Never smoker | 748 (75%) | 49 (63%) |  | 769 (63%) | 126 (64%) |  |
| Former smoker | 80 (9%) | 10 (10%) |  | 160 (18%) | 23 (18%) |  |
| Current smoker | 136 (16%) | 19 (27%) |  | 202 (19%) | 43 (18%) |  |
| **History of pelvic infection** |  |  | 0.12 |  |  | 0.46 |
| Yes | 30 (3%) | 4 (7%) |  | 63 (5%) | 19 (7%) |  |
| No | 930 (97%) | 74 (93%) |  | 1,060 (95%) | 172 (93%) |  |
| **Regular periods** |  |  | 0.12 |  |  | 0.33 |
| Yes | 916 (96%) | 72 (92%) |  | 1,054 (94%) | 180 (91%) |  |
| No | 48 (4%) | 6 (8%) |  | 77 (6%) | 12 (9%) |  |
| **Depression status** |  |  | 0.29 |  |  | 0.33 |
| Yes | 94 (10%) | 15 (14%) |  | 109 (10%) | 24 (14%) |  |
| No | 868 (90%) | 63 (86%) |  | 1,021 (90%) | 168 (86%) |  |
| **Cotinine (ng/mL)** | 31 ± 82 | 56 ± 97 | **<0.05** | 45 ± 108 | 43 ± 111 | 0.59 |
| **Calories (kcal/d)** | 1,841 ± 643 | 1,913 ± 758 | 0.66 | 1,853 ± 631 | 1,778 ± 624 | 0.34 |
| **Sleeplessness** |  |  | 0.70 |  |  | **<0.05** |
| Yes | 174 (23%) | 16 (27%) |  | 288 (26%) | 70 (37%) |  |
| No | 790 (77%) | 62 (73%) |  | 843 (74%) | 122 (63%) |  |

Continuous variables were presented as weighted mean ± standard deviation and categorical variables were presented as unweighted frequency and percentage. Bold indicated statistical significance. *P* value in bold indicated statistical significance.

Abbreviations: BMI: body mass index; PIR: poverty-income ratio; DII: dietary inflammation index.

Table S3. Characteristics of the participants based on BMI subgroup analysis.

| **Variables** | **< 25 kg/m^2^** | | | **25 to < 30 kg/m^2^** | | | **≥ 30 kg/m^2^** | | |
| --- | --- | --- | --- | --- | --- | --- | --- | --- | --- |
|  | **Control**  **(N = 14,908,232)** | **Infertility**  **(N = 1,387,076)** | ***P* value** | **Control**  **(N = 9,549,248)** | **Infertility**  **(N = 982,281)** | ***P* value** | **Control**  **(N = 14,803,499)** | **Infertility**  **(N = 2,737,357)** | ***P* value** |
| **Age(years)** | 30 ± 7 | 35 ± 6 | **<0.001** | 31 ± 7 | 34 ± 6 | **0.04** | 32 ± 7 | 33 ± 6 | 0.14 |
| **Race/Ethnicity** |  |  | 0.76 |  |  | 0.88 |  |  | 0.61 |
| Mexican American | 71 (7%) | 7 (4%) |  | 110 (15%) | 9 (14%) |  | 162 (15%) | 33 (18%) |  |
| Other Hispanic | 82 (9%) | 11 (9%) |  | 58 (7%) | 7 (11%) |  | 82 (8%) | 14 (7%) |  |
| Non-Hispanic White | 230 (60%) | 26 (62%) |  | 150 (56%) | 12 (50%) |  | 229 (49%) | 46 (53%) |  |
| Non-Hispanic Black | 134 (10%) | 16 (14%) |  | 109 (11%) | 9 (12%) |  | 290 (20%) | 38 (15%) |  |
| Non-Hispanic Asian | 158 (11%) | 16 (9%) |  | 67 (7%) | 8 (6%) |  | 39 (3%) | 6 (2%) |  |
| Other/ multiracial | 32 (3%) | 1 (2%) |  | 21 (4%) | 2 (7%) |  | 57 (5%) | 9 (5%) |  |
| **Education** |  |  | 0.34 |  |  | **0.03** |  |  | 0.59 |
| Less than high school | 73 (7.0%) | 9 (13%) |  | 92 (9.7%) | 10 (12%) |  | 123 (10%) | 22 (12%) |  |
| High school | 116 (18%) | 19 (23%) |  | 104 (21%) | 7 (7%) |  | 192 (24%) | 31 (26%) |  |
| More than high school | 518 (75%) | 49 (64%) |  | 319 (70%) | 30 (81%) |  | 544 (66%) | 93 (62%) |  |
| **Marriage** |  |  | **<0.001** |  |  | 0.24 |  |  | **<0.05** |
| Married/ Living with partner | 360 (52%) | 56 (81%) |  | 309 (62%) | 36 (74%) |  | 437 (54%) | 104 (72%) |  |
| Widowed/ Divorced/ Separated | 45 (5%) | 9 (8%) |  | 46 (11%) | 5 (15%) |  | 90 (9%) | 16 (12%) |  |
| Never married | 302 (42%) | 12 (11%) |  | 160 (27%) | 6 (10%) |  | 332 (37%) | 26 (16%) |  |
| **PIR** | 2.83 ± 1.70 | 3.01 ± 1.53 | 0.46 | 2.81 ± 1.73 | 3.77 ± 1.58 | **0.01** | 2.50 ± 1.60 | 2.39 ± 1.53 | 0.52 |
| **Sedentary behavior** |  |  | 0.25 |  |  | 0.46 |  |  | 0.85 |
| Mild | 489 (71%) | 56 (78%) |  | 361 (67%) | 26 (59%) |  | 577 (65%) | 91 (64%) |  |
| Severe | 218 (29%) | 21 (22%) |  | 154 (33%) | 21 (41%) |  | 282 (35%) | 55 (36%) |  |
| **Physical activity** |  |  | 0.18 |  |  | **0.01** |  |  | **0.03** |
| Light | 374 (44%) | 52 (55%) |  | 297 (48%) | 34 (73%) |  | 578 (64%) | 92 (50%) |  |
| Vigorous/Moderate | 333 (56%) | 25 (45%) |  | 218 (52%) | 13 (27%) |  | 281 (36%) | 54 (50%) |  |
| **DII** | 0.31 ± 2.11 | 1.02 ± 1.78 | **0.02** | 0.45 ± 2.08 | 0.38 ± 1.97 | 0.66 | 0.85 ± 1.81 | 1.01 ± 1.95 | 0.31 |
| **Drinking status** |  |  | 0.20 |  |  | 0.59 |  |  | 0.30 |
| Never drinker | 289 (36%) | 31 (43%) |  | 229 (43%) | 15 (34%) |  | 309 (34%) | 57 (35%) |  |
| Former drinker | 192 (25%) | 25 (35%) |  | 131 (19%) | 21 (26%) |  | 303 (33%) | 57 (40%) |  |
| Current drinker | 226 (40%) | 21 (23%) |  | 155 (39%) | 11 (40%) |  | 247 (32%) | 32 (25%) |  |
| **Smoking status** |  |  | 0.12 |  |  | 0.83 |  |  | 0.22 |
| Never smoker | 534 (71%) | 51 (66%) |  | 385 (69%) | 34 (75%) |  | 589 (67%) | 90 (59%) |  |
| Former smoker | 71 (12%) | 4 (5.7%) |  | 55 (12%) | 5 (10%) |  | 113 (15%) | 24 (22%) |  |
| Current smoker | 102 (16%) | 22 (29%) |  | 75 (18%) | 8 (15%) |  | 157 (18%) | 32 (19%) |  |
| **History of pelvic infection** |  |  | 0.21 |  |  | 0.43 |  |  | 0.42 |
| Yes | 21 (1%) | 5 (3%) |  | 22 (5%) | 3 (9%) |  | 50 (6%) | 15 (8%) |  |
| No | 680 (99%) | 72 (97%) |  | 490 (95%) | 44 (91%) |  | 806 (94%) | 130 (92%) |  |
| **Regular periods** |  |  | **<0.05** |  |  | 0.08 |  |  | 0.83 |
| Yes | 666 (96%) | 69 (82%) |  | 485 (94%) | 45 (98%) |  | 805 (94%) | 138 (93%) |  |
| No | 41 (4%) | 8 (18%) |  | 30 (6%) | 2 (2%) |  | 54 (6%) | 8 (7%) |  |
| **Depression status** |  |  | >0.05 |  |  | 0.72 |  |  | >0.99 |
| Yes | 46 (7.3%) | 10 (18%) |  | 53 (12%) | 6 (15%) |  | 104 (12%) | 23 (12%) |  |
| No | 658 (93%) | 67 (82%) |  | 462 (88%) | 41 (85%) |  | 755 (88%) | 123 (88%) |  |
| **Cotinine (ng/mL)** | 40 ± 105 | 99 ± 157 | 0.59 | 41 ± 97 | 33 ± 84 | 0.25 | 35 ± 87 | 28 ± 75 | 0.38 |
| **Calories (kcal/d)** | 1,815 ± 629 | 1,722 ± 618 | 0.53 | 1,855 ± 572 | 1,941 ± 708 | 0.38 | 1,875 ± 682 | 1,826 ± 677 | 0.41 |
| **Sleeplessness** |  |  | 0.72 |  |  | 0.90 |  |  | **0.04** |
| Yes | 125 (19%) | 17 (23%) |  | 102 (24%) | 18 (23%) |  | 230 (30%) | 51 (44%) |  |
| No | 582 (81%) | 60 (77%) |  | 413 (76%) | 29 (77%) |  | 629 (70%) | 95 (56%) |  |

Continuous variables were presented as weighted mean ± standard deviation and categorical variables were presented as unweighted frequency and percentage. Bold indicated statistical significance. *P* value in bold indicated statistical significance.
